# Autofluorescence Profiling of Virgin Olive Oil: Impact of Rosemary and Basil Flavoring During Storage

**DOI:** 10.3390/antiox15010062

**Published:** 2026-01-01

**Authors:** Enrique J. Díaz-Montaña, Ramón Aparicio-Ruiz, Noelia Tena, Ana Lobo-Prieto, Diego L. García-González, María Teresa Morales

**Affiliations:** 1 Departamento de Química Analítica, Facultad de Farmacia, Universidad de Sevilla, Prof. García González, 2, 41012 Sevilla, Spainntena@us.es (N.T.);; 2 Instituto de la Grasa (IG), CSIC, Ctra. de Utrera, km. 1, Edificio 46, 41013 Sevilla, Spain

**Keywords:** flavored virgin olive oil, autofluorescence, oxidation, aromatic plants, green analytical chemistry

## Abstract

The consumption of virgin olive oil has been associated with a broad spectrum of beneficial effects. These health outcomes are attributed not only to its high monounsaturated fatty acid content but also to its bioactive components. Nowadays, the flavoring of olive oil has gained popularity to improve its antioxidant properties, modify its sensory characteristics, and enhance its oxidative stability. This study explores spectrofluorometry as a fast, non-destructive, and eco-friendly tool to monitor oxidation and predict shelf life in virgin olive oils (VOOs). Both unflavored and flavored rosemary and basil samples were studied. Over nine months of storage, monthly autofluorescence measurements at 330 nm excitation revealed dynamic spectral changes. These changes were mapped into three distinct emission zones (I, II, and III), providing a spectral fingerprint of oil freshness and stability. Autofluorescence analysis revealed that oxidation-related emission increased while pigment-related emission decreased over time, especially within the first five months. Rosemary and basil flavoring slowed degradation due to antioxidant migration from the herbs. It is proposed that a ratio between the fluorescence intensity of Zone III/Zone II of the spectrum of less than 0.6 indicates oils stored for more than three months.

## 1. Introduction

Virgin olive oil (VOO) is a central component of the Mediterranean diet and is widely valued for its unique organoleptic, nutritional, and health-related properties. Its consumption has been associated with a broad spectrum of beneficial effects, including the prevention of cardiovascular diseases, type 2 diabetes, metabolic syndrome, and various forms of cancer [1,2]. These health outcomes are attributed not only to its high content of monounsaturated fatty acids—primarily oleic acid—but also to its rich profile of bioactive minor components, such as phenolic compounds, tocopherols (vitamin E), phytosterols, and pigments (chlorophylls and carotenoids) [3,4,5]. These minor compounds, though present at low concentrations, exert antioxidant, anti-inflammatory, and antimicrobial activities and play a pivotal role in protecting the oil from oxidative degradation while enhancing its biological value [6].

The stability and quality of virgin olive oil are highly dependent on both intrinsic factors—such as cultivar, ripeness of the olives, and processing techniques—and extrinsic factors such as storage conditions (light, oxygen, and temperature). Oxidative processes lead to the gradual deterioration of oil quality over time, manifesting as the loss of key volatile and phenolic compounds and the generation of undesirable off-flavors and rancid notes [7,8]. These chemical and sensory changes ultimately define the shelf life of the product and its classification within regulatory standards. In addition, the stability and quality of virgin olive oil can be further modified through emerging practices such as the flavoring of the oil with herbs and spices, a trend gaining increasing attention for its potential to modify sensory attributes and enhance functional properties of the virgin olive oil.

Nowadays, the flavoring oils have gained popularity, and it is possible to find flavored olive oils, among others, obtained by incorporating aromatic herbs such as rosemary (*Rosmarinus officinalis* L.) and basil (*Ocimum basilicum* L.). These plants are rich in polyphenols and essential oils with potent antioxidant properties, which can migrate into the oil matrix during storage [9,10,11]. Previous studies have demonstrated that these additions can modify the sensory characteristics of the oil while enhancing its oxidative stability [11,12,13]. However, there is a lack of knowledge about how incorporating aromatic herbs influences the oxidative stability of virgin olive oil over time, especially in long-term storage or using non-destructive techniques.

Several analytical methods are currently employed to monitor olive oil oxidation, including peroxide value, UV absorbance (K_232_ and K_270_), and sensory evaluation. These techniques can be labor-intensive, time-consuming, and require the use of solvents or trained personnel. There is a growing interest in developing rapid, non-destructive, and environmentally sustainable techniques to evaluate the quality and oxidative stability of VOO during storage [7,14]. In this regard, fluorescence spectroscopy is a powerful analytical tool, capable of providing detailed molecular-level information on the chemical composition of oils without the need for sample preparation or derivatization.

Several minor components of virgin olive oil, such as tocopherols, chlorophylls, pheophytins, and phenolic compounds, naturally exhibit luminescent properties that can be effectively exploited using spectrofluorometric techniques. In addition to these native antioxidants, various oxidation products, including conjugated dienes and trienes, also display intrinsic fluorescence [15,16,17]. Each of these compound classes emits at characteristic wavelengths when excited at specific excitation wavelengths, contributing to the oil’s overall autofluorescence spectrum. Moreover, when virgin olive oil is flavored with aromatic herbs such as rosemary (*Rosmarinus officinalis* L.) and basil (*Ocimum basilicum* L.), additional fluorescent compounds—such as eugenol, linalool, carnosic acid, and rosmarinic acid—can migrate into the oil matrix [18,19]. These plant-derived molecules not only enhance the antioxidant potential of the oil but also contribute to its autofluorescence signature, potentially enriching the spectral information available for quality assessment. This spectral profile is expected to evolve over time as oxidation progresses, potentially offering a molecular-level fingerprint of the oil’s chemical status and freshness. In this context, the autofluorescence spectra of virgin olive oil may serve as a non-destructive spectroscopic indicator of its quality and oxidative state—an aspect that this study aims to investigate and validate.

The use of fluorescence spectroscopy in food analysis has expanded significantly in recent years, driven by its high sensitivity, selectivity, low cost, and minimal environmental impact [20]. It has found broad application in fields such as molecular biology, clinical diagnostics, environmental monitoring, and food safety. In the case of olive oil, both steady-state and time-resolved fluorescence measurements have been employed to monitor oxidation, degradation kinetics of natural antioxidants and pigments during storage and thermal stress, assess authenticity, and detect adulteration [21,22]. However, a thorough review of the scientific literature reveals a lack of studies specifically addressing the use of this technique in flavored virgin olive oils, particularly those infused with aromatic herbs. This highlights the need for innovative tools capable not only of assessing the oxidative status of VOO but also of capturing the modulatory effects of herbal flavoring on its oxidative degradation process. In this context, autofluorescence spectroscopy emerges as a promising approach, offering a holistic view of chemical transformations during storage and enabling the identification of fluorescent markers indicative of oil aging and oxidative progression. This research stands out by combining autofluorescence to profile herb-flavored virgin olive oils with an assessment of their oxidative evolution under commercial storage conditions.

Thus, this study explores autofluorescence spectroscopy as a novel, non-invasive, and sustainable technique for monitoring oxidation and shelf life in virgin olive oil (VOO), including both unflavored and herb-flavored samples. The research aims to identify specific emission zones and spectral ratios that serve as reliable markers of freshness and degradation. Additionally, the influence of rosemary and basil flavoring on oxidative stability is assessed to determine whether these herbs alter the fluorescence profile during storage. Through the integration of autofluorescence and chemometric analysis, this work proposes a rapid and eco-friendly methodology for real-time quality control in the olive oil industry, with potential applications in product traceability, consumer protection, and environmental stewardship. Thus, the study contributes novel insights into the potential of autofluorescence as a rapid, non-destructive tool for quality monitoring in a growing segment of the olive oil market.

## 2. Materials and Methods

### 2.1. Samples and Flavoring Procedure

A commercial extra virgin olive oil (EVOO) of the Manzanilla variety, harvested during the 2023/2024 season and purchased from a local market in Seville, Spain, was selected as the control sample (coded as AC). This control sample was divided into three sets: one set of non-flavored oil and two sets of flavored oils. The two flavored oils control sets were prepared following previous studies [11] by adding 5% (*w*/*w*) of dried rosemary (*Rosmarinus officinalis* L.) (coded as ROO) and basil (*Ocimum basilicum* L.) (coded as BOO) leaves, respectively. The aromatic plants were harvested from the Seville countryside (37.320471 N, −5.979523 W), Andalusia, Spain, in September 2023, manually separated from woody parts, and dried at 60 °C for 10 h [23].

Additionally, four validation samples (TS1–TS4) purchased in a local market in Seville from the 2023/2024 season and different cultivars—Hojiblanca (TS1), Arbequina (TS2, TS4), and Picual (TS3)—were included to evaluate the broader applicability of the method.

All olive oil samples—both flavored and unflavored—were packaged in 100 mL transparent glass containers. A total of seven sample types were prepared: AC (unflavored control olive oil), ROO (rosemary-flavored olive oil), BOO (basil-flavored olive oil), TS1 (Hojiblanca validation sample), TS2 (Arbequina validation sample), TS3 (Picual validation sample), and TS4 (Arbequina validation sample). For each sample type, nine identical containers were prepared, resulting in a total of 63 individual units for analysis.

### 2.2. Storage Conditions

The samples (63 samples from AC, ROO, BOO, TS1, TS2, TS3, and TS4) were stored under controlled conditions simulating typical commercial environments (15.7 ± 3.6 °C and 12 h/day of light (500 ± 100 lx) for a total period of nine months. From the moment the plant material was placed in contact with the oil, the samples were immediately stored under the experimental conditions detailed in the methodology. Each month, fluorescence analyses were conducted.

### 2.3. Fluorescence Spectroscopy

Autofluorescence measurements were performed using a Shimadzu RF-1501 spectrofluorometer (Shimadzu Corporation, Kyoto, Japan) equipped with a 150 W xenon lamp, monochromators, and PC-controlled acquisition software (PC-150x). Emission spectra were recorded in triplicate over the 350–850 nm range using excitation wavelengths (λ_ex_) of 330, 350, and 370 nm during method optimization. Based on signal intensity and repeatability, λ_ex_ = 330 nm was selected for all measurements.

Samples (3 mL) were transferred into 10 mm pathlength non-fluorescent optical glass cuvettes (Hellma 101-0S (Hellma, Müllheim, Germany)). Each cuvette was cleaned between samples using sequential rinses of hexane, methanol, Extran^®^ detergent, distilled water, and acetone, followed by drying with filtered air.

### 2.4. Quality Parameters and Sensory Assessment

The quality parameters defined in the international trade standard for olive oils and olive-pomace oils [24] were assessed to verify the time-0 quality of AC, TS1-TS4 virgin olive oil samples, following the corresponding standard methods: peroxide value (PV) [25], free acidity (free fatty acids or FFA) [26], ultraviolet absorbance at 270 nm and 232 nm (K_270_ and K_232_ indexes, respectively) [27], and fatty acid composition [28]. The organoleptic assessment [29] was conducted on non-stored samples to confirm the absence of sensory defects in the collected virgin olive oils before starting the incubation experiment. Other parameters of interest related to these samples were also determined, such as total phenol content by the Folin–Ciocalteau method [30] and oxidative stability index (OSI) by the Rancimat method [31]. All analyses were performed in triplicate.

### 2.5. Statistical Analysis

Spectral data were exported to Microsoft Excel for initial processing. Statistical analyses were performed using STATISTICA 8.0 (StatSoft Inc., Tulsa, OK, USA). Additionally, Savitzky–Golay smoothing, as well as setting the baseline to zero, was applied. The coefficient of variation was employed to evaluate the repeatability (CVr) and the intermediate precision (CVR) of the fluorescence method across selected spectral zones. Differences in fluorescence intensity across treatments and storage times were assessed by one-way ANOVA followed by post hoc analysis. Brown–Forsythe tests were used to select significant wavelengths for classification. Principal component analysis (PCA) and linear discriminant analysis (LDA) were applied to investigate group separation and classification accuracy based on autofluorescence profiles.

## 3. Results and Discussion

### 3.1. Characterization of Virgin Olive Oil Samples

The quality category of the non-stored (time 0) virgin olive oil samples (AC, TS1–TS4) was established based on the physicochemical and sensory quality parameters, determined according to the IOC standard [24]. The categorization is of great interest to know the quality characteristics of the samples at the beginning of the study. Therefore, free acidity, peroxide value, extinction coefficients at 232 and 270 nm, and organoleptic properties were determined (Table 1). Other parameters of interest related to these samples were also determined, such as total phenol content by the Folin–Ciocalteau method [30] and the oxidative stability index by the OSI Rancimat method (Table 1) [31].

The evaluation of the physicochemical quality parameters confirmed that all the olive oil samples, except TS4, could be classified in the extra virgin category at the beginning of the study, since they did not exceed the limits established by the regulations. Organoleptic evaluation, prior to the storage experiment, showed sensory differences between the samples. The assessors detected a green fruity odor in the control sample (AC) and an intense green and fruity odor in the TS1, which explained the higher mean value of the fruity sensory attribute. The TS3 extra virgin olive oil was characterized by a high median of the fruity attribute and of the bitter and pungent attributes, typical of the Picual variety. On the other hand, oils TS2 and TS4, of the Arbequina variety, showed a delicate fruitiness with slightly bitter and spicy notes, showing the lowest median of the fruity attribute. The sensory quality parameters (Table 1) of sample TS4, due to a low intensity of the winey-vinegary sensory defect (1.9), led to its assignment to the virgin category.

The stability of the oils was also evaluated using the Rancimat method, the sample TS3 showing the highest stability (Table 1) with a value of 82.80 h, which is in agreement with its higher concentration of total phenols, antioxidant compounds responsible for protecting the oil against oxidation. Sample TS2 presented the lowest value with 22.95 h, even though it did not present the lowest phenol content.

### 3.2. Wavelength Selection for Autofluorescence Measurements

A study was carried out to select the adequate λ_ex_ for autofluorescence measurements. Based on this purpose, the emission spectra of the control virgin olive oil sample (AC) were recorded at three λ_ex_, 330, 350 and 370 nm, and, to select the best measurement conditions, a precision study was carried out considering both repeatability conditions (six replicates/single operator/one work session) and intermediate precision (two operators/five work sessions).

The precision study was carried out considering three different zones of the emission spectra of virgin olive that are related to the concentration of different fluorophore compounds present in the sample [32]. In Supplementary Materials, Figure S1 represents the emission spectrum of the control virgin olive oil (AC) at λ_ex_ = 330 nm, where three zones of the spectrum (I, II, and III), corresponding to the intervals 360–420 nm, 420–620 nm, and 620–720 nm, have been marked. In the spectrum, the areas with a λ_em_ below and far away from the λ_ex_ have been discarded. Different authors [32,33,34,35,36] have related zone I of the spectrum to the concentration of tocopherols, tocotrienols, and phenolic compounds, zone II to the formation of fluorophore compounds during oil oxidation and the presence of conjugated dienes and trienes, and zone III to the concentration of pigments, mainly chlorophylls and pheophytins in virgin olive oils.

The precision values obtained for the spectrum acquired at the different wavelengths selected for the method (λ_ex_ = 330, 350, and 370 nm) are shown in Table 2. The statistical parameters used to express both repeatability and intermediate precision were the standard deviation (SD) and the coefficient of variation (CV), with CV values below 10% being acceptable. Therefore, Table 2 shows the values obtained for the standard deviation, as well as for the coefficients of variation in both cases and in each of the three zones indicated in the spectrum.

The results shown in Table 2 indicate very good repeatability, with values for the coefficients of variation below 10% in all cases for λ_ex_ = 330, whereas for the other two wavelengths, it was over the 10% limit. As for intermediate precision, the CVR values (%) were higher than those obtained in the repeatability evaluation, due to the greater variability introduced. These results are similar to those obtained in other studies [35] and demonstrate the good precision of this type of method. Furthermore, λ_ex_ = 330 was the only wavelength that did not saturate the instrumental signal. Thus, based on the precision of the results obtained and the intensity of the signal, which did not show saturation in any area of the spectrum and which allowed evaluation of all the changes produced during the study, 330 nm was selected as the most suitable λ_ex_ to carry out the study of the shelf life of flavored and unflavored virgin olive oils.

### 3.3. Autofluorescence Spectral Analysis of the Control Virgin Oil (AC) During Storage

**Figure 1:**
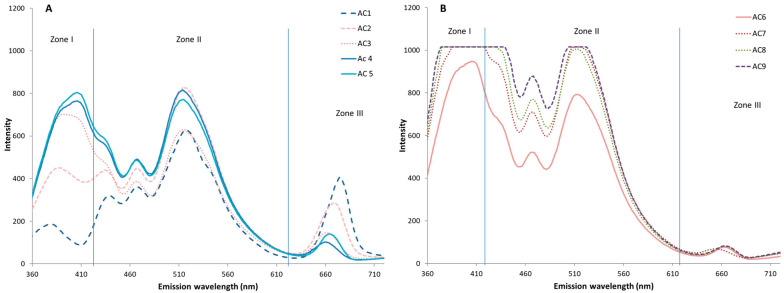
Emission spectra of the control virgin olive oil (AC) during storage from 0 to 5 months (**A**) and from 6 to 9 months (**B**), with the three studied zones (I, II, III).

Throughout the study, the autofluorescence spectra of the control virgin oil sample (AC) were acquired every month during the nine months of storage (Figure 1). Intensity variations occurred in the three zones of the spectrum, but these variations were positively correlated with time in the case of zones I and II, where the fluorescence intensity increased during the storage period, and negatively correlated with storage time in zone III, where the emission intensity of the bands decreased. The changes occurred most dramatically during the first five months of storage, while during the remaining four months the spectrum changed more slowly. These changes could be caused by the formation of oxidation compounds during the storage period (zones I and II), which justifies the increase in intensity, and by a decrease in chlorophyll pigments (zone III), compounds that degraded during storage, which is in agreement with the results obtained by other authors [32,34].

The modifications detected in the spectrum showed that the emission spectrum of virgin olive oil at a λ_ex_ = 330 nm contains relevant information about its oxidation state and can be used to evaluate the shelf life of olive oils, as already described by other authors using different types of fluorescence [21,37].

### 3.4. Comparative Study of Autofluorescence Spectra in Flavored and Unflavored Virgin Olive Oils

The emission spectra of the rosemary- and basil-flavored olive oil samples were recorded under the same conditions as those used for the unflavored oil. The emission spectrum of the flavored and unflavored oils, after one month of storage, showed the same profile, from a qualitative point of view, presenting the three characteristic zones. Although the unflavored oil reached higher intensity values than the flavored oil, the application of an ANOVA to the data of the first month samples did not reveal significant differences (*p* < 0.05) between them, probably because during this first month of storage, the contact time was not sufficient for the effect of the presence of the aromatic plants on the oil to be observed.

**Figure 2:**
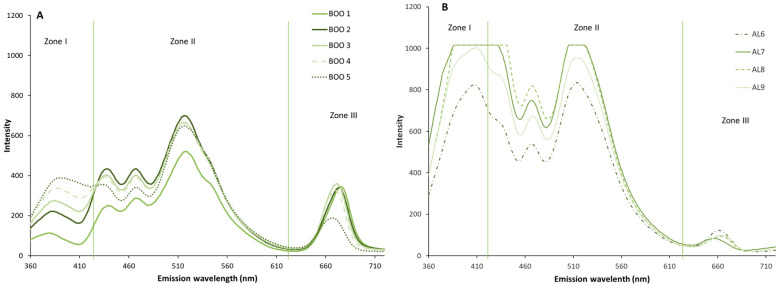
Emission spectra of basil-flavored oil (BOO) during storage from 0 to 5 months (**A**) and from 6 to 9 months (**B**), with the three studied zones (I, II, III).

**Figure 3:**
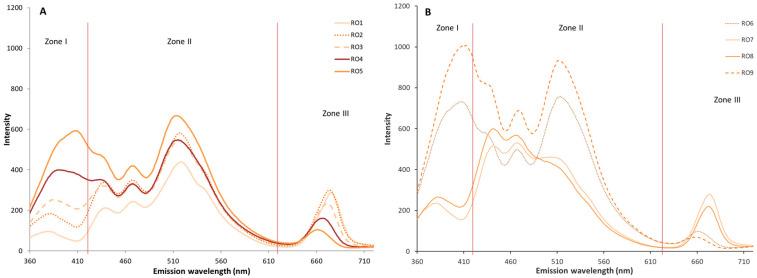
Emission spectra of rosemary-flavored oil (ROO) during storage from 0 to 5 months (**A**) and from 6 to 9 months (**B**), with the three studied zones (I, II, III).

As in the unflavored olive oil sample, the evolution of the flavored oils was studied during the nine months of storage. As can be seen in Figure 2 and Figure 3, corresponding to rosemary and basil, respectively, there are variations in the intensity and/or in the areas of the spectrum. These variations occur in the same direction as in the control oil, such as increases in fluorescence intensity during storage in zones I and II, and a decrease in zone III. The intensity of the bands was lower in both cases than in the control oil, especially during the first months of storage (Figure 2A and Figure 3A), indicating a slower evolution of oxidation, possibly due to a migration of antioxidant compounds from the plant material to the oil. This delay in the appearance of oxidation compounds, inferred from the fluorescence trend, has been described by other authors, who observed how the migration of antioxidant compounds from plants can improve the oxidative stability of vegetable oils [38].

The spectra corresponding to 6–9 months of storage (Figure 2B and Figure 3B) also reached high values of fluorescence intensity in the presence of the plants during storage, but the values reached were below those of the control oil (AC) in many areas of the spectrum.

Similarly, to the unflavored control oil, an ANOVA was performed considering the fluorescence intensity of the flavored oils, and the results showed that storage time has a significant effect (*p* < 0.05) on the variation of the emission spectrum. The ANOVA showed that storage time significantly (*p* < 0.05) affects all wavelengths of the spectrum, except for the 643–648 nm range of BOO samples.

In the samples studied, a decrease in the zone III band was observed throughout storage, corresponding to chlorophyll pigments and derivatives. This decrease in fluorescence intensity was attributed to oxidative processes. The effects of light and temperature lead to the degradation of chlorophylls into pheophytins and, subsequently, into non-colored compounds, as has been demonstrated by other authors [39].

**Figure 4:**
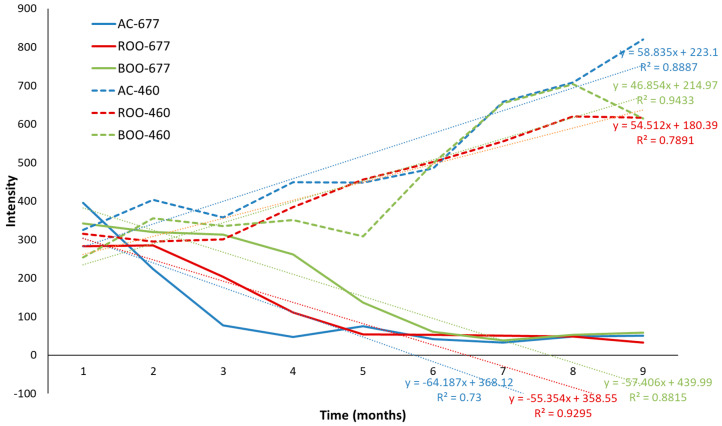
Plot of the average intensity of zone II (458–462; dot-line) and zone III (675–679 nm; line) versus time in control unflavored virgin olive oil (AC; blue) and flavored with rosemary (ROO; red) and basil (BOO; green) samples.

When plotting the average emission intensity around the maximum of zone III (675–679 nm) over time, a progressive decline was observed across all three oil matrices. Notably, the decreasing rate was greater in the unflavored control sample, showing the highest slope in terms of absolute value (64.18), suggesting a lower resistance to oxidative degradation in the absence of plant-derived antioxidants. In contrast, rosemary- and basil-flavored oils exhibited a slower decline in fluorescence intensity, particularly during the early stages of storage. From the sixth month onward, the emission intensities of all samples converged to similar levels, indicating that the protective effect of the added herbs is most pronounced during the initial months of storage (Figure 4). Additionally, a time-dependent red shift in the emission maximum was detected, consistent with the progressive degradation of chlorophylls into pheophytins and, eventually, into non-fluorescent compounds. This spectral shift further supports the role of autofluorescence as a sensitive indicator of pigment transformation and oxidative progression in virgin olive oil. Additionally, in Figure 4, the average intensity around the maximum of zone II, 458–462 nm, was also plotted, and the behavior was the opposite of that described for zone III, showing an increase in intensity related to the generation of oxidation compounds. The lower intensities in the first months indicate the protective role of plants. As for zone III, this cannot be observed after the sixth month of storage. The unflavored virgin olive oil sample showed the highest oxidation rate and the highest slope (58.35), confirming, as in zone III, the protective role of the plants. However, the behavior did not fully fit a linear model in any of the plotted areas, with determination coefficients ranging from 0.7891 to 0.9433 for zone II and from 0.7300 to 0.9295 for zone III.

**Figure 5:**
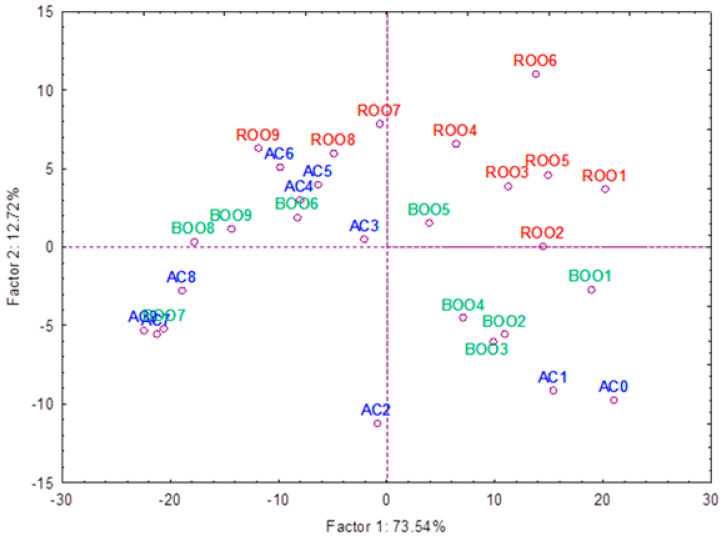
Principal component analysis (PCA) of the control sample (AC) and the flavored samples (BOO and ROO) using the information of the full spectrum.

Once the differences between flavored and unflavored samples were found, the potential of the spectral information to differentiate between the two types of samples as a function of storage time was evaluated. A principal component analysis was applied using as variables the intensities obtained at all λ_em_ of the spectra (Figure 5). The results revealed a clear and consistent separation between fresh and stored samples, highlighting the significant impact of storage on olive oil quality across both flavored and unflavored oils (Figure 5). The spatial distribution of the samples along the principal components indicates that rosemary and basil not only modify the oxidative behavior of the oils but also confer distinct spectral signatures that persist throughout storage. PC1, that explains a 73.54% of the variance, is related to time-dependent progression: fresher samples (AC-1 and AC-2; BOO-1 to BOO-5; ROO-1 to ROO-6) cluster on the positive side of the axis, while longer-stored, more oxidized samples shift to the negative side, underscoring the protective role of the herbs that reach the freshness until month 5 or 6. PC2, which explains 12.72% of the variance, further differentiates the oils, with most rosemary- and basil-flavored samples clustering in the positive region, while the control (AC) and a few basil-flavored oils appear in the negative region. From the sixth month onward, however, the distinction between groups diminishes, reflecting the loss of the protective effect of the herbs and the convergence of all oils toward similar oxidative states, as observed previously.

To investigate potential differences between the stored flavored and unflavored olive oil samples, a series of statistical analyses was performed on the emission spectral data. Following the application of ANOVA and principal component analysis (PCA) across the full spectral range, a Brown–Forsythe test was conducted to identify specific wavelengths showing statistically significant differences (*p* < 0.05) between flavored and unflavored oils. Only wavelengths within zone I were selected—corresponding to the emission region associated with tocopherols and phenolic compounds, which are key antioxidant constituents in olive oil (Table 3). This finding suggests that zone I is particularly sensitive to the presence of flavoring agents, likely due to the enhanced antioxidant content in the flavored samples. As oxidation progresses, these compounds are gradually depleted, but their initial higher concentration in the flavored oils may explain the observed spectral differences and support the use of this region as a discriminative marker for flavorization effects.

**Figure 6:**
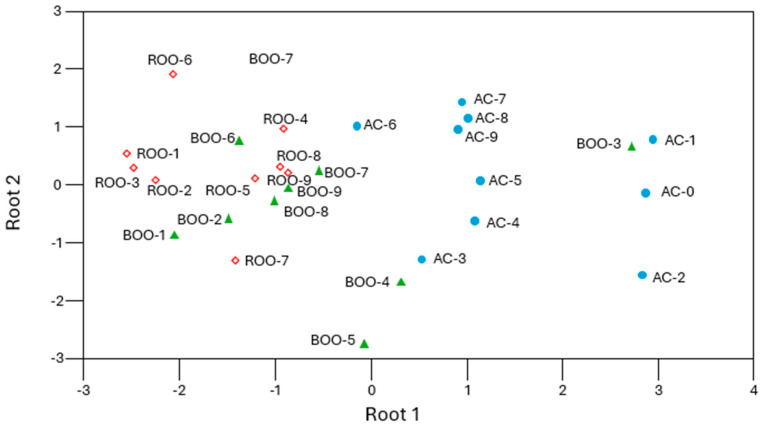
Separation of control (AC, blue circle), rosemary-flavored (ROO, red rhomb), and basil-flavored (BOO, green triangle) samples based on data selected by the Brown–Forsythe test using linear discriminant analysis.

Finally, a linear discriminant analysis (LDA) was performed using the emission intensities at the wavelengths identified by the Brown–Forsythe test. The model achieved a correct classification rate of 71%, indicating that most of the samples were accurately assigned to their respective groups, flavored or unflavored, based on their spectral profiles. The wavelengths with the highest discriminative power were 361, 365, 366, 368, and 369 nm (Figure 6), all located within zone I, which is associated with antioxidant compounds such as tocopherols and phenolics. The LDA plot (Figure 6) revealed a clear separation between flavored and unflavored oils, supporting the hypothesis that the presence of herbal additives significantly alters the fluorescence signature of the oils. Although complete separation was not achieved, due to overlapping effects of storage time, the formation of two distinct clusters confirms that autofluorescence spectra contain sufficient chemical information to differentiate between oil types.

These findings demonstrate, for the first time, that autofluorescence spectroscopy combined with multivariate statistical tools can not only monitor oxidative changes but also discriminate between flavored and unflavored virgin olive oils.

### 3.5. Development and Validation of Predictive Models for Olive Oil Degradation Based on Autofluorescence Spectra

To further investigate the observed differences and assess the reproducibility of the results, an additional set of samples (TS1–TS4) was incorporated as a validation set. This set of validation samples was analyzed following the same methodology as the control and flavored samples, evaluating their quality parameters (Table 1) and performing spectrofluorimetric analyses every month during the storage period.

The autofluorescence spectra of the validation samples exhibited similar profiles and spectral features to those observed in the control oil, with variations in the progression of oxidative changes depending on the specific characteristics of each sample. To explore the relationship between spectral changes and storage time, a correlation analysis was performed using the emission intensities across different spectral zones of the control oil. Distinct regions showed either positive or negative correlations with time. The strongest positive correlations were found in the ranges of λ_em_ = 390–424 nm (r = 0.90–0.94) and λ_em_ = 550–600 nm (r = 0.90–0.92), while a strong negative correlation was observed in the λ_em_ = 620–720 nm region (r = –0.81 to –0.92). These results are consistent with the known degradation pathways of fluorescent compounds in olive oil: the decline in pigment-related signals (e.g., chlorophylls and pheophytins) and the concurrent increase in the fluorescence intensity from oxidation-derived compounds.

**Figure 7:**
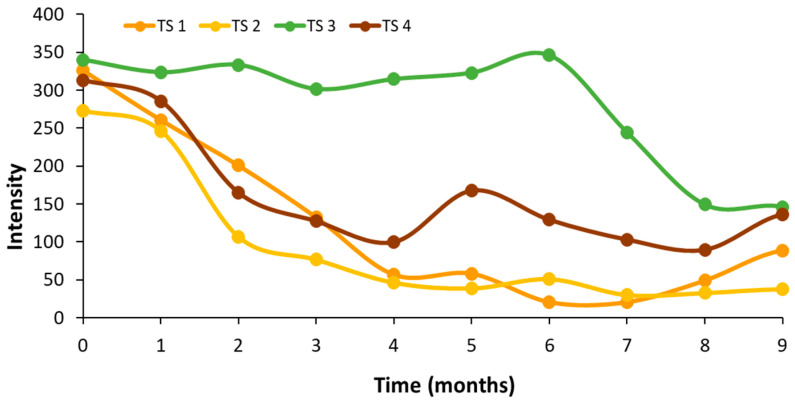
Average intensity of the maximum of the band of the zone III (675–679 nm) of the validation samples versus storage time.

Figure 7 shows the average emission intensity obtained for the validation samples around the maximum of zone III (675–679 nm). A trend similar to that of the control sample (Figure 4) can be observed, with an appreciable decrease in signal intensity until the fourth month and a subsequent stabilization, with the Picual *var*. sample (TS3) taking the longest to reach low intensity values, which may be justified because it is the sample that at time zero presented the highest oxidative stability index (Rancimat), the highest concentration of total phenols and the lowest values of K_232_ and K_270_.

**Figure 8:**
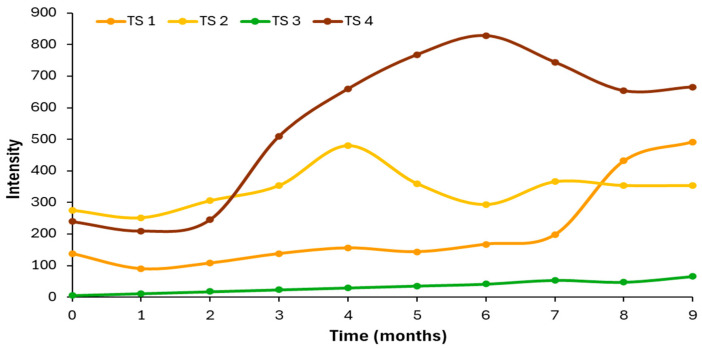
Average intensity of the maximum of the band of zone II (458–462 nm) of the validation samples versus storage time.

On the other hand, Figure 8 shows the average emission intensity values obtained for the validation samples around the maximum of zone II (458–462 nm), which corresponds to the zone of oxidation compounds, hence the increase that occurs in signal intensity with storage time, which has been previously described by other authors [40].

In this case, sample TS3 is the one that presented the smallest increase in the intensity of this band, precisely due to what has been explained above. Samples TS1 and TS2 showed intermediate values, and sample TS4, which had initially been categorized as virgin, on the basis of the regulated quality parameters, showed the greatest increase in intensity.

These results further support the potential of autofluorescence spectroscopy to sensitively monitor the chemical evolution of different types of virgin olive oils during storage, using common emission bands as reliable indicators of oxidative progression.

**Figure 9:**
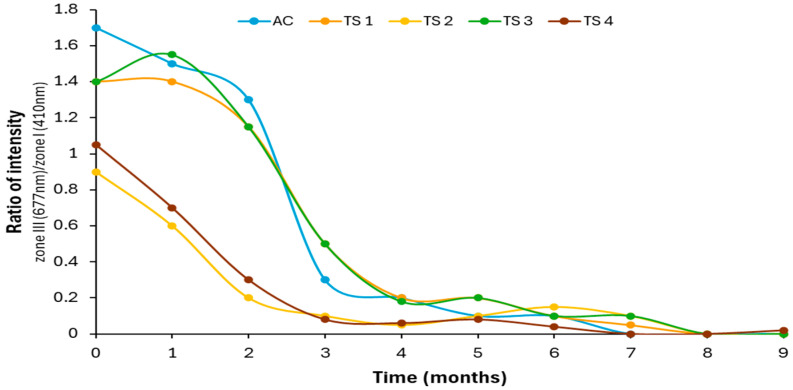
Ratio of the average intensity around the maximum of the band of zone III (675–679 nm) between that of zone II (458–462 nm) of the control and validation samples versus storage time.

To propose a predictive model linking oil degradation over time with autofluorescence behavior, the ratio between the average emission intensities around the maximum of zones III and II was calculated. Figure 9 displays the results obtained using the peak emission wavelengths of each zone. The data reveal a clear downward trend in this ratio, with a steeper decline observed during the first three months of storage, followed by a continued but slower decrease that eventually stabilizes in the final months.

This trend reflects the progressive degradation of pigments relative to antioxidant compounds and supports the preliminary use of the Zone III/Zone II emission intensity ratio as a potential spectroscopic marker of oxidative status, at least under the experimental conditions applied in this study. Based on the results obtained, a threshold value of <0.6 is proposed for this ratio to indicate advanced oxidation. In contrast, oils with less than three months of storage consistently showed values above 0.6, suggesting that this index could serve as a reliable indicator of freshness. Given its consistency across all studied samples and its basis in the behavior of intrinsic fluorophores in virgin olive oil, this ratio holds promise as a practical tool for monitoring oil quality over time. Further studies are encouraged to validate its applicability across different cultivars, processing conditions, and storage environments, with the aim of establishing a robust, non-destructive method for assessing freshness and shelf life in commercial olive oils.

## 4. Conclusions

This study demonstrates the applicability of autofluorescence spectroscopy as a reliable, non-destructive, and green analytical approach for evaluating the oxidative status and shelf life of virgin olive oils. The autofluorescence emission spectra, particularly when acquired at λ_ex_ = 330 nm, reflect specific changes in the chemical composition of the oil during storage, including the degradation of pigments and the accumulation of oxidation products. The definition of three key emission zones enabled the monitoring of critical molecular events associated with oil aging.

The incorporation of rosemary and basil as flavoring agents resulted in a measurable delay in oxidative degradation, particularly evident during the early stages of storage. This protective effect was evident in the fluorescence patterns, suggesting that autofluorescence can serve not only to evaluate oil freshness but also to assess the efficacy of natural antioxidants introduced via herbal flavoring.

A novel spectral marker—the ratio of the emission intensities of pigments to oxidation products (Zone III/Zone II)—was proposed as an indicator of oil oxidative state. A threshold value of 0.6 was identified, below which oils were consistently classified as having been stored for more than three months.

The proposed method demonstrated robustness across different olive oil cultivars, with multivariate analysis significantly enhancing its classification performance. Collectively, these results support the adoption of autofluorescence spectroscopy as a practical and sustainable tool for routine quality control and freshness assessment in the olive oil sector.

To strengthen and standardize the use of fluorescence-based markers, future research should expand this approach to include a wider variety of olive oil types, processing techniques, and packaging formats. Such efforts will be essential to validate the generalizability of the method and to establish reliable spectroscopic indicators for industrial applications.

## Figures and Tables

**Table 1:** Quality parameters (organoleptic evaluation, K_232_, K_270_, peroxide value, and free acidity) according to IOC standards [24], oxidative stability index by Rancimat, total phenols content, and category of the virgin olive oil samples at time 0.

Quality Parameter		AC	TS1	TS2	TS3	TS4
Organoleptic characteristics	Median of defect	0.0 ± 0.0	0.0 ± 0.0	0.0 ± 0.0	0.0 ± 0.0	1.9 ± 0.2
	Median of fruity	3.7 ± 0.2	4.7 ± 0.3	3.5 ± 0.3	3.8 ± 0.2	3.0 ± 0.3
Physicochemical parameters	K_232_ ($K_{1\mathrm{cm}}^{1\%}$)	1.49 ± 0.07	1.52 ± 0.08	1.93 ± 0.08	0.98 ± 0.4	1.84 ± 0.7
	K_270_ ($K_{1\mathrm{cm}}^{1\%}$)	0.19 ± 0.01	0.06 ± 0.00	0.15 ± 0.01	0.03 ± 0.00	0.18 ± 0.01
	Peroxide value (meq O_2_/kg)	8.0 ± 0.3	4.3 ± 0.1	6.5 ± 0.2	2.4 ± 0.1	4.8 ± 0.2
	Free acidity (% p/p oleic acid)	0.50 ± 0.02	0.11 ± 0.01	0.22 ± 0.01	0.11 ± 0.00	0.22 ± 0.01
Other characteristics	Rancimat oxidative stability index (100 °C)	41.00 ± 0.9	38.71 ± 0.8	22.95 ± 0.7	82.80 ± 0.9	53.70 ± 0.8
	Total phenols (mg gallic acid equivalents/kg)	323 ± 9	270 ± 5	340 ± 6	590 ± 8	420 ± 6
Category		AOVE	AOVE	AOVE	AOVE	AOV

**Table 2:** Standard deviation and coefficient of variation (%) of each zone of the emission spectrum at λ_ex_ = 330, 350, and 370 nm under repeatability (SDr and CVr) and intermediate precision (SDR and CVR) conditions.

Excitation Wavelength (λ_ex_ nm)	330				350				370			
Spectrum Zone (λ_em_ nm)	SDr	CVr	SDR	CVR	SDr	CVr	SDR	CVR	SDr	CVr	SDR	CVR
Zone I (360–420 nm)	17.71	5.53	34.11	8.92	19.47	5.70	37.64	11.07	24.13	6.72	39.32	11.56
Zone II (420–620 nm)	25.41	6.81	34.75	9.87	32.69	8.22	-	-	-	-	-	-
Zone III (620–720 nm)	1.64	2.85	5.45	9.27	2.35	3.61	6.02	9.31	4.01	5.72	7.12	10.17

Note: - refers to saturated areas where no signal could be recorded.

**Table 3:** Emission wavelengths showing significant differences (*p* < 0.05) between flavored and non-flavored samples according to the Brown–Forsythe test.

Emission Wavelength (nm)	F	*p*
361	4.703999	0.012655
362	4.573701	0.014166
363	4.469833	0.015505
364	4.295164	0.018057
365	4.127271	0.020921
366	4.055566	0.022284
367	3.992487	0.023559
368	3.840128	0.026957
369	3.681781	0.031030
370	3.356536	0.041515

## Data Availability

The original contributions presented in this study are included in the article and Supplementary Materials. Further inquiries can be directed to the corresponding author.

## Supplementary Materials

The following supporting information can be downloaded at https://www.mdpi.com/article/10.3390/antiox15010062/s1. Figure S1. Emission spectrum of the control VOO sample (AC) (λ_ex_ = 330 nm) with the three differentiated zones (zone I related to tocopherols, tocotrienols, and phenolic compounds; zone II compounds generated during oil oxidation and the presence of conjugated dienes and trienes, and zone III to the concentration of pigments).
