# Supplementary material for: Autofluorescence Profiling of Virgin Olive Oil: Impact of Rosemary and Basil Flavoring During Storage
Source: Antioxidants (Basel). 2026 Jan 1;15(1):62. doi: 10.3390/antiox15010062 (PMC12837548; doi:10.3390/antiox15010062)
Supplement: Supplementary file 1 [file antioxidants-15-00062-s001.zip › antioxidants-4036609-Supplementary Material.pdf]

Supplementary Material

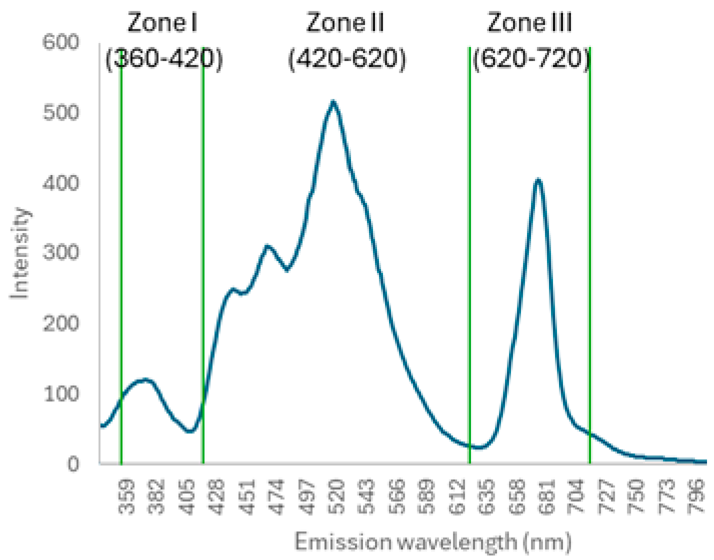


**Figure S1.** Emission spectrum of the control VOO sample (AC) ($\lambda_{ex}$ = *330 nm)* with the three differentiated zones (zone I related to tocopherols, tocotrienols and phenolic compounds; zone II compounds generated during oil oxidation and the presence of conjugated dienes and trienes, and zone III to the concentration of pigments).
